# Mental Health and Information Reporting Assistant: technological innovation including low- and middle-income countries - an update

**DOI:** 10.1192/j.eurpsy.2022.1456

**Published:** 2022-09-01

**Authors:** R. Zimmermann

**Affiliations:** Univerity of Basel, Faculty For Psychology, Basel, Switzerland

**Keywords:** digital health, Early detection, LMIC, evidence-based assessment

## Abstract

**Introduction:**

According to the World Health Organization, addressing the mental health care gap for adolescents, especially in low-resource contexts, is a priority. Evidence-based assessment is crucial for selecting treatment strategies and for quality management.

**Objectives:**

To develop a digital platform for evidence-based assessments and implement it in different low-resource settings.

**Methods:**

The project operates according to the principles of digital development (https://digitalprinciples.org/), including designing with the user, user testing, understanding the ecosystem, resusing software and being open source, think about sustainability and addressing privacy and security.

**Results:**

Different implementation contexts (in Tanzania, Kosovo and Chile) will be presented.

The learned lessons will be presented to the audience.

**Conclusions:**

MHIRA is a promising tool that helps bridge the gap regarding adolescent mental health in low-resource settings. Challenges include the clinicans attitude towards evidence based assessment, sustainability of the project and integration with the existing information technology eco-system and regulations.

**Disclosure:**

No significant relationships.

